# Transarterial Infusion Chemotherapy with FOLFOX Could be an Effective and Safe Treatment for Unresectable Intrahepatic Cholangiocarcinoma

**DOI:** 10.1155/2022/2724476

**Published:** 2022-03-15

**Authors:** Shaohua Li, Min Deng, Qiaoxuan Wang, Jie Mei, Jingwen Zou, Wenping Lin, Ming Shi, Minshan Chen, Wei Wei, Rongping Guo

**Affiliations:** ^1^Department of Liver Surgery, Sun Yat-Sen University Cancer Center, Guangzhou, China; ^2^State Key Laboratory of Oncology in South China, Collaborative Innovation Center for Cancer Medicine, Guangzhou, China; ^3^Department of Radiation Oncology, Sun Yat-Sen University Cancer Center, Guangzhou, China

## Abstract

**Background:**

Transarterial infusion (TAI) chemotherapy with the FOLFOX regimen has shown good efficacy and safety in the treatment of hepatocellular carcinoma (HCC). However, it has not been reported in intrahepatic cholangiocarcinoma (ICC).

**Methods:**

The data of consecutive patients with unresectable ICC who underwent TAI with the FOLFOX regimen from November 2016 to September 2019 were retrospectively analyzed. Treatment effectiveness and safety were evaluated and compared using the Kaplan–Meier method, log-rank test, Cox regression models, and *χ*^2^ test.

**Results:**

Twenty-nine patients were included in the study. The median overall survival (OS) was 16.2 months (95% CI, 13.0–19.4). The median progression-free survival (PFS) was 8.7 months (95% CI, 6.2–11.1). Twenty-seven patients were included in the efficacy analysis. There were 0, 10, 13, and 4 patients with CR, PR, SD, and PD, respectively, based on mRECIST criteria. The ORR was 37.0%, and the DCR was 85.2%. There were 27 patients (93.1%) who experienced grade 1-2 AEs, while only 1 patient experienced grade 3 AEs.

**Conclusion:**

TAI with the FOLFOX regimen could be an effective and safe treatment for unresectable ICC.

## 1. Introduction

Intrahepatic cholangiocarcinoma (ICC) is the second most common primary liver cancer and has an increasing incidence and mortality rate [1, 2]. The optimal treatment for ICC is surgical resection. However, only 12% of patients with ICC have localized disease at presentation owing the infiltrative nature of this disease [3]. ICC that cannot be surgically resected has poorer prognosis and more controversial treatment options.

Currently, the commonly used nonsurgical liver-directed local treatments for ICC include transarterial chemoembolization (TACE), transarterial infusion (TAI) chemotherapy, and transarterial radioembolization (TARE). TACE achieves the purpose of treatment by intra-arterial injection of an emulsion of lipiodol or chemotherapeutic drugs to occlude the tumor feeding artery. At present, it is considered to be the standard for intermediate stage HCC [4]. In TAI, a high concentration chemotherapeutic agent is injected into the liver through the hepatic artery, so the concentration of the drug at the tumor site can improve the antitumor effect [5]. TARE is used to treat tumors by delivering radioactive materials to the tumor artery for local irradiation [6]. However, the vast majority of the studies have been retrospective, and the number of cases enrolled in each study has been relatively small, resulting in considerably questionable results.

TAI chemotherapy with the FOLFOX regimen was found to improve the prognosis of patients with intermediate and advanced hepatocellular carcinoma (HCC) [7–9]. TAI has been associated with a high tumor objective response rate (ORR), satisfactory local lesion control, and a low incidence of adverse events (AEs) in our previous studies. Herein, we performed a retrospective trial to determine the efficacy and safety of TAI chemotherapy in ICC patients.

## 2. Material and Methods

### 2.1. Trial Design

This is a retrospective study that was conducted at the Sun Yat-Sen University Cancer Center (Guangzhou, China). The trial protocol was approved by the Institutional Review Board (IRB) and Institutional Ethics Committee (IEC) of Sun Yat-Sen University Cancer Center and complied with the Declaration of Helsinki and good clinical practice guidelines. All patients involved provided written informed consent for participation.

### 2.2. Eligibility Criteria

We followed the methods of Guo et al. [10]. The eligibility criteria for inclusion were as follows: 18 years or older and 75 years or younger; Eastern Cooperative Oncology Group Performance Score (ECOG-PS) ≤2; histologically confirmed ICC not suitable for curative treatments including surgery, transplantation, ablation, and radiotherapy; no previous treatment for ICC; and adequate hematologic, hepatic, and renal functions (absolute neutrophil (NE) count ≥1.5 × 10^9^/L, hemoglobin (Hgb) ≥80 g/L, platelet (PLT) count ≥60 × 10^9^/L, serum albumin (ALB) ≥28 g/L, serum total bilirubin (TBil) ≤3× the upper limit of normal, serum aspartate transaminase (AST) and alanine transaminase (ALT) ≤5× the upper limit of normal, serum creatinine (CRE) ≤1.5× the upper limit of normal, prothrombin time (PT) ≤19.5 seconds, and international standardized ratio of prothrombin (INR) ≤2.3). Patients were fully informed and provided signed informed consent forms.

The exclusion criteria were as follows: severe functional impairment of important organs such as the heart, brain, lung, kidney, and liver; allergy to related drugs or intolerance to TAI treatment; history of other malignancies; pregnancy, breastfeeding, or lack of use of adequate contraception among women with childbearing potential; history of organ transplantation; neurological or psychiatric diseases that may affect cognitive assessment and informed consent; previous or concomitant antitumor therapy including interferon or participation in other interventional clinical trials; history of esophageal or gastric variceal bleeding, hepatic encephalopathy, or cardio cerebrovascular events within 30 days of treatment; medical history of human immunodeficiency virus (HIV) infection; drug addiction or drug abuse; and other factors that may have affected patient enrollment and assessment of obtained results.

### 2.3. Treatment Procedures

TAI procedures were performed as described in our previous reports [7, 8, 11]. Briefly, a femoral artery puncture was performed using Seldinger's technique, and catheterization was performed routinely. A 5 French vascular puncture device and a 5 French catheter were routinely used in TAI. The microcatheter was not routinely used by all patients. A 2.6 French microcatheter will be used for patients with difficult catheter placement and apparent vascular variation. The catheter/microcatheter was placed into the tumor's main feeding hepatic artery, and the following regimen was performed through the hepatic artery: the TAI regimen comprised oxaliplatin 135 mg/m^2^ from hour 0 to 3 on day 1; leucovorin 400 mg/m^2^ from hour 3 to 4.5 on day 1; fluorouracil, 400 mg/m^2^ from hour 4.5 to 6.5 on day 1; and fluorouracil, 2400 mg/m^2^ over 46 hours from day 1 to day 3. The catheter and microcatheter were removed together when TAI was completed. Repetitive catheterization was performed in the next TAI cycle. TAI was performed once every 3 to 4 weeks. Any implanted port system was not applied.

### 2.4. Follow-Up Evaluation and Survival Analyses

Each follow-up session included history taking, physical examination, laboratory tests, and contrast-enhanced computed tomography (CT) or/and magnetic resonance imaging (MRI) examination. The initial follow-up appointment was 6 to 8 weeks (2 cycles) after TAI was performed. The primary endpoint was overall survival (OS), which was defined as the time from TAI until death from any cause. The secondary endpoints included progression-free survival (PFS), tumor response rate, and treatment safety. PFS was defined as the time from treatment until disease progression or death, whichever came first. The tumor response rate included the objective response rate (ORR) and disease control rate (DCR). ORR was defined as the percentage of patients achieving either complete response (CR) or partial response (PR), which needed to be maintained for at least 4 weeks from the first radiological confirmation. DCR was defined as the ORR plus the percentage of patients with stable disease (SD). Tumor response was evaluated according to the mRECIST criteria [12]. Adverse events were graded according to the National Cancer Institute Common Terminology Criteria for Adverse Events (NCI-CTCAE) version 5.0 [13]. This study was censored on May 27, 2020.

### 2.5. Statistical Analysis

Categorical variables were compared using Pearson's *χ*^2^ test or Fisher's exact test. Variable distributions were described using the mean ± standard error (SE) for normally distributed values and the median and range for non-normally distributed values. Continuous variables were compared using Student's *t*-test for normally distributed values or Mann–Whitney test for skewed values. Survival analyses were performed using Kaplan–Meier method, and differences in the survival curves were analyzed using the log-rank test. A two-tailed *p* < 0.05 was considered statistically significant.

All analyses were performed according to the intention-to-treat (ITT) principle. All data analyses were performed using the SPSS software, version 24.0 (SPSS Inc., Chicago, IL, USA).

## 3. Results

### 3.1. Patient Characteristics and Treatment

Between November 2016 and September 2019, 29 patients who met the inclusion criteria were included in this study. All included patients were treated in the Department of Liver Surgery, Sun Yat-Sen University Cancer Center. The clinical characteristics of the patients are shown in Table 1. All included patients were treated with a total of 92 cycles (median 3 cycles, range 1–6 cycles) of TAI.

### 3.2. Efficacy Analysis

**Figure 1:**
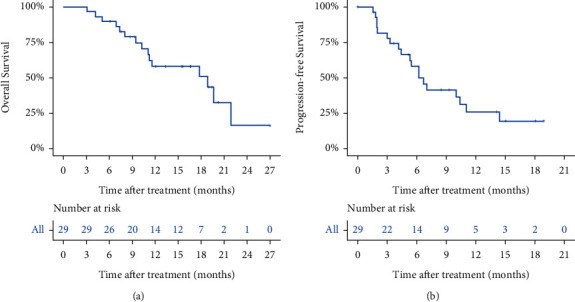
Survival analysis of patients. (a) OS of patients. (b) PFS of patients.

By May 27, 2020, with a median follow-up time of 11.6 months (range, 3.1 to 27.0 months), 15 patients (51.7%) had died. The median OS was 16.2 months (95% CI, 13.0–19.4). Nineteen patients (65.5%) had disease progression. The median PFS was 8.7 months (95% CI, 6.2–11.1) (Figure 1).

**Figure 2:**
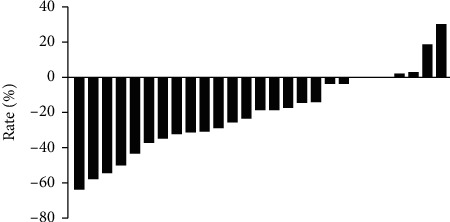
Efficacy analysis of the 27 patients. The histograms show the rate of change of maximum tumor diameter according to mRECIST during FOLFOX chemotherapy.

There were 27 patients in whom efficacy could be evaluated. There were 0, 10, 13, and 4 patients with CR, PR, SD, and PD, respectively, based on mRECIST criteria [12] (Figure 2). The ORR was 37.0%, and the DCR was 85.2%. There were 2, 3, and 1 patient who had intrahepatic, extrahepatic, and macrovascular tumor thrombosis progression, respectively.

Univariate and multivariate Cox regression analyses for OS and PFS showed that age ≤50 years was the only independent prognostic indicator for both OS and PFS (Tables S1 and S2).

**Figure 3:**
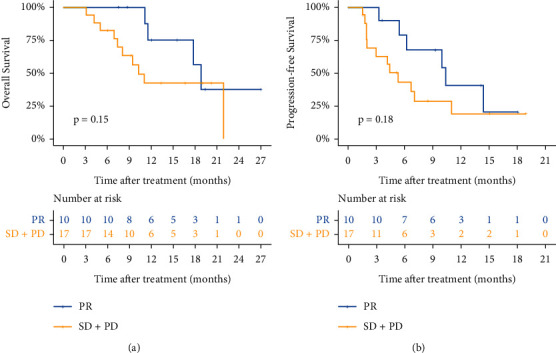
Survival analysis of patients with tumor response and nonresponders. (a) OS of patients with tumor response and nonresponders. (b) PFS of patients with tumor response and nonresponders.

The clinical characteristics of patients with tumor response (*n* = 10) and nonresponders (*n* = 17) (Table 2) showed that neutrophil count and serum CA19-9 level were lower in patients with tumor response than those in nonresponders. However, the serum AFP level and HBV-DNA level were higher in patients with tumor response than those in nonresponders. The median OS of patients with tumor response was 18.8 months (95% CI, 16.7–20.9), and that for nonresponders was 10.3 months (95% CI, 7.4–13.1) (*p*=0.148, Figure 3(a)). The median PFS of patients with tumor response was 10.4 months (95% CI, 5.1–15.7), and that for nonresponders was 5.3 months (95% CI, 3.2–7.5) (*p*=0.177, Figure 3(b)).

After TAI, the patients underwent subsequent antitumor therapies, including TACE, radiofrequency ablation (RFA), immune checkpoint inhibitor (ICI) treatment, targeted therapy (including apatinib and lenvatinib), chemotherapy, radiotherapy, and surgical operation (Table S3).

### 3.3. Safety Analysis

There were 27 patients (93.1%) who experienced grade 1-2 AEs, while only 1 patient experienced grade 3 AEs. All AEs were mild and manageable, and no toxicity-associated deaths occurred in the present study. Anorexia, anemia, elevated ALT levels, hypoalbuminemia, hyperbilirubinemia, pain, and vomiting were the most common AEs (Table S4).

## 4. Discussion

At present, few approaches have proven effective for unresectable ICC, showing unsatisfactory survival benefits. We have achieved some success through the treatment of HCC with TAI with the FOLFOX regimen [7–9, 11]. However, for ICC, which is generally considered to have a lack of arterial blood supply, there is a lack of data from prospective clinical trials. Therefore, we retrospectively analyzed existing clinical data to evaluate the efficacy and safety of TAI with the FOLFOX regimen for ICC and lay the foundation for further prospective clinical trials.

Based on the results of the present study, patients with ICC who underwent TAI with the FOLFOX regimen had an ORR of 37.0% and a DCR of 85.2%. The mPFS and mOS were 8.7 and 16.2 months, respectively. The results showed that the efficacy of TAI with the FOLFOX regimen for ICC was reliable. TAI with the FOLFOX regimen also showed good safety in the safety analysis. Especially in the absence of embolic agents, compared with TACE, TAI caused milder inflammatory reactions and bile duct injury, reducing the risk of liver failure and bile duct-related complications, which have been reported as the main causes of death in patients with ICC [3, 14]. In addition, a recent study on the efficacy of interventional therapy for unresectable ICC showed that HAIC was superior to TACE [15]. The safety difference between TAI and TACE was also consistent with that in our previous reports [11, 16].

The good safety and moderate treatment intensity of TAI with the FOLFOX regimen mean that it is a perfect option for the multiagent treatment of ICC, which benefits from a combination of targeted therapy, ICI therapy, radiotherapy, and other treatment options.

Moreover, in our unpublished study, the ORR of TAI with the FOLFOX regimen for advanced HCC was 44.3%, and the mOS was 27.8 months, suggesting that TAI may be superior to TACE in the treatment of advanced HCC. Interestingly, although all of our patients were confirmed to have ICC by puncture pathology, the patients with higher AFP and HBV-DNA levels seemed to have a better response to TAI treatment than those with lower AFP and HBV-DNA levels, suggesting that in real-world clinical practice, a considerable proportion of ICCs confirmed by puncture pathology are actually mixed cell carcinomas; similarly, and the more HCC components, the better the response to TAI with the FOLFOX regimen will be.

There are several limitations of the present study. First, this is a retrospective study with inherent biases. Second, the number of cases included in the present study is relatively small. A large-scale prospective randomized clinical trial is needed to further confirm the efficacy and safety of TAI with the FOLFOX regimen in ICC in the future.

In conclusion, the present study demonstrated that TAI with the FOLFOX regimen could be an effective and safe treatment for unresectable ICC. However, the results need to be further confirmed through large-scale prospective randomized clinical trials in the future.

## Figures and Tables

**Table 1:** Clinical characteristics of patients.

	*n* = 29
Age (year)	51.4 (28.0 – 67.0)
Sex	
Male	21 (72.4%)
Female	8 (27.6%)
WBC count (×10^9^/L)	7.8 ± 0.5
NE count (×10^9^/L)	5.4 ± 0.5
Hgb (g/L)	133.6 ± 4.0
PLT count (×10^9^/L)	258.6 ± 21.1
ALT (U/L)	25.15 (7.2 – 64.2)
ALB (g/L)	42.3 ± 0.8
TBil (umol/L)	13.0 ± 1.0
PT (s)	12.1 ± 0.1
CRE (umol/L)	68.7 ± 3.1
AFP (ng/ml)	4.5 (1.14 – 18,596.0)
CA19-9 (U/ml)	58.9 (0.6 – 20,000.0)
CEA (ng/ml)	3.9 (1.02 – 169.8)
HBsAg	
Negative	10 (34.5%)
Positive	19 (65.5%)
Anti-HCV	
Negative	29 (100.0%)
Positive	0 (0.0%)
HBV-DNA	
≤1 × 10^3^ copies	15 (51.7%)
>1 × 10^3^ copies	14 (48.3%)
Child–Pugh score	
5	28 (96.6%)
6	1 (3.4%)
Maximum diameter of tumor (cm)	8.4 ± 0.6
Tumor numbers	
Single	12 (41.4%)
Multiple	17 (58.6%)
Tumor distribution	
Unilobe	18 (62.1%)
Bilobe	11 (37.9%)
Macrovascular invasion	
Absent	20 (69.0%)
Present	9 (31.0%)
Distant metastasis	
Absent	18 (62.1%)
Present	11 (37.9%)

**Table 2:** Clinical characteristics of patients with tumor response and nonresponders.

	Responders (*n* = 10)	Nonresponder (*n* = 17)	*p* value
Age (year)	56 (28.0 – 67.0)	52 (32.0 – 60.0)	0.269
Sex			0.204
Male	9 (90.0%)	11 (64.7%)	
Female	1 (10.0%)	6 (35.3%)	
WBC count (×10^9^/L)	7.5 ± 0.5	7.9 ± 0.7	0.093
NE count (×10^9^/L)	5.2 ± 0.5	5.5 ± 0.7	0.033
Hgb (g/L)	131.1 ± 5.6	136.4 ± 5.6	0.629
PLT count (×10^9^/L)	260.9 ± 35.0	253.1 ± 29.6	0.483
ALT (U/L)	25.2 (14.8 – 53.8)	23.3 (11.0 – 64.2)	0.514
ALB (g/L)	41.6 ± 1.7	42.7 ± 0.9	0.250
TBil (umol/L)	13.8 ± 1.8	13.5 ± 1.4	0.999
PT (s)	12.4 ± 0.3	12.1 ± 0.2	0.156
CRE (umol/L)	75.6 ± 4.9	65.2 ± 3.7	0.967
AFP (ng/ml)	9.5 (2.7 – 18,596.0)	4.2 (1.1 – 91.7)	0.035
CA19-9 (U/ml)	17.85 (0.6 – 384.2)	154.0 (0.7 – 20,000.0)	0.016
CEA (ng/ml)	3.0 (1.0 – 169.8)	5.4 (1.1 – 63.3)	0.874
HBsAg			0.406
Negative	2 (20.0%)	7 (41.2%)	
Positive	8 (80.0%)	10 (58.8%)	
HBV-DNA			0.018
≤1 × 10^3^ copies	2 (20.0%)	12 (70.6%)	
>1 × 10^3^ copies	8 (80.0%)	5 (29.4%)	
Child–Pugh score			0.370
5	9 (90.0%)	17 (100.0%)	
6	1 (10.0%)	0 (0.0%)	
Maximum diameter of tumor (cm)	9.1 ± 1.1	8.0 ± 0.7	0.460
Tumor numbers			0.257
Single	6 (60.0%)	6 (35.3%)	
Multiple	4 (40.0%)	11 (64.7%)	
Tumor distribution			0.219
Unilobe	5 (50.0%)	13 (76.5%)	
Bilobe	5 (50.0%)	4 (23.5%)	
Macrovascular invasion			0.415
Absent	6 (60.0%)	13 (76.5%)	
Present	4 (40.0%)	4 (23.5%)	
Distant metastasis			0.230
Absent	8 (80.0%)	9 (52.9%)	
Present	2 (20.0%)	8 (47.1%)	

## Data Availability

The data that support the findings of our study are available upon request from the corresponding author. The data are not publicly available due to privacy or ethical restrictions.

## Supplementary Materials

Supplementary Materials Table S1: univariate and multivariate Cox regression analysis for OS. Table S2: univariate and multivariate Cox regression analysis for PFS. Table S3: subsequent antitumor therapies prescribed to the patients. Table S4: treatment related adverse events (AEs) of patients.
