# Supplementary material for: Transarterial Infusion Chemotherapy with FOLFOX Could be an Effective and Safe Treatment for Unresectable Intrahepatic Cholangiocarcinoma
Source: J Oncol. 2022 Mar 15;2022:2724476. doi: 10.1155/2022/2724476 (PMC8941539; doi:10.1155/2022/2724476)
Supplement: Supplementary Materials — Table S1: univariate and multivariate Cox regression analysis for OS. Table S2: univariate and multivariate Cox regression analysis for PFS. Table S3: subsequent antitumor therapies prescribed to the patients. Table S4: treatment related adverse events (AEs) of patients. [file 2724476.f1.docx]

**Table of contents:**

Table S1. Univariate and multivariate Cox regression analysis for OS.

Table S2. Univariate and multivariate Cox regression analysis for PFS.

Table S3. Subsequent antitumor therapies prescribed to the patients.

Table S4. Treatment related adverse events (AEs) of patients.

Table S1. Univariate and multivariate Cox regression analysis for OS.

|  | Univariate Cox regression analysis for OS | | | Multivariate Cox regression analysis for OS | | |
| --- | --- | --- | --- | --- | --- | --- |
| Variables | HR | 95% CI | p value | HR | 95% CI | p value |
| Male | 2.511 | 0.560-11.263 | 0.229 |  |  |  |
| Age≤50 yr | 3.145 | 0.939-10.532 | 0.063 | 3.936 | 1.119-13.842 | 0.033 |
| Multiple lesions | 1.286 | 0.430-3.851 | 0.653 |  |  |  |
| Bilobe involved | 1.120 | 0.387-3.244 | 0.835 |  |  |  |
| Maximum diameter≤8 cm | 1.315 | 0.455-3.798 | 0.613 |  |  |  |
| Macrovascular invasion absent | 1.150 | 0.390-3.395 | 0.800 |  |  |  |
| Metastasis present | 1.797 | 0.644-5.011 | 0.263 |  |  |  |
| CA19-9 > 35U/ml | 1.908 | 0.604-6.021 | 0.271 |  |  |  |
| CEA >5 ng/ml | 2.592 | 0.873-7.696 | 0.086 | 2.557 | 0.834-7.836 | 0.100 |
| AFP >25 ng/ml | 1.507 | 0.501-4.531 | 0.465 |  |  |  |
| ALT>40 U/L | 2.295 | 0.798-6.602 | 0.123 |  |  |  |
| ALB>40 g/L | 1.222 | 0.382-3.908 | 0.735 |  |  |  |
| HBsAg negative | 1.679 | 0.539-5.236 | 0.372 |  |  |  |
| HBV DNA≤1×10^3^ copies | 1.807 | 0.578-5.651 | 0.309 |  |  |  |
| CA 19-9 not decrease after TAI | 1.318 | 0.440-3.946 | 0.622 |  |  |  |
| CEA decrease after TAI | 2.480 | 0.691-8.899 | 0.164 |  |  |  |
| AFP decrease after TAI | 2.423 | 0.721-8.136 | 0.152 |  |  |  |
| Tumor non-responder | 2.309 | 0.719-7.418 | 0.160 |  |  |  |

Table S2. Univariate and multivariate Cox regression analysis for PFS.

|  | Univariate Cox regression analysis for OS | | | Multivariate Cox regression analysis for OS | | |
| --- | --- | --- | --- | --- | --- | --- |
| Variables | HR | 95% CI | p value | HR | 95% CI | p value |
| Male | 1.770 | 0.585-5.356 | 0.312 |  |  |  |
| Age≤50 yr | 2.551 | 0.970-6.713 | 0.058 | 3.591 | 1.006-12.817 | 0.049 |
| Multiple lesions | 2.598 | 0.957-7.053 | 0.061 | 2.782 | 0.863-8.970 | 0.087 |
| Unilobe involved | 1.521 | 0.547-4.235 | 0.422 |  |  |  |
| Maximum diameter>8 cm | 1.337 | 0.536-3.335 | 0.534 |  |  |  |
| Macrovascular invasion absent | 1.333 | 0.474-3.753 | 0.586 |  |  |  |
| Metastasis present | 1.202 | 0.481-3.003 | 0.694 |  |  |  |
| CA19-9 > 35U/ml | 1.957 | 0.726-5.279 | 0.185 |  |  |  |
| CEA >5 ng/ml | 2.484 | 0.939-6.570 | 0.067 | 2.697 | 0.928-7.838 | 0.068 |
| AFP >25 ng/ml | 1.549 | 0.549-4.367 | 0.408 |  |  |  |
| ALT≤40 U/L | 1.026 | 0.334-3.151 | 0.964 |  |  |  |
| ALB>40 g/L | 1.151 | 0.410-3.230 | 0.789 |  |  |  |
| HBsAg positive | 1.317 | 0.499-3.476 | 0.578 |  |  |  |
| HBV DNA≤1×10^3^ copies | 1.104 | 0.445-2.737 | 0.831 |  |  |  |
| CA19-9 not decrease after TAI | 1.990 | 0.779-5.084 | 0.151 |  |  |  |
| CEA decrease after TAI | 2.705 | 0.966-7.580 | 0.058 | 1.827 | 0.563-5.925 | 0.316 |
| AFP decrease after TAI | 1.052 | 0.407-2.720 | 0.916 |  |  |  |

Table S3. Subsequent antitumor therapies prescribed to the patients.

| Treatment | n |
| --- | --- |
| TACE | 3 |
| RFA | 1 |
| ICI | 10 |
| Apatinib | 3 |
| Lenvatinib | 5 |
| Chemotherapy | 1 |
| Radiotherapy | 3 |
| Surgical operation | 1 |

Table S4. Treatment related adverse events (AEs) of patients.

| AEs and grade | N (%) |
| --- | --- |
| Overall |  |
| 0 | 1 (3.4) |
| 1-2 | 27 (93.1) |
| 3-4 | 1 (3.4) |
| Anorexia |  |
| 0 | 22 (75.9) |
| 1 | 4 (13.8) |
| 2 | 3 (10.3) |
| Anemia |  |
| 0 | 15 (51.7) |
| 1 | 10 (34.5) |
| 2 | 3 (10.3) |
| 3 | 1 (3.4) |
| ALT level elevated |  |
| 0 | 25 (86.2) |
| 1 | 3 (10.3) |
| 2 | 1 (3.4) |
| Neutropenia |  |
| 0 | 27 (93.1) |
| 1 | 1 (3.4) |
| 2 | 1 (3.4) |
| Hypoalbuminemia |  |
| 0 | 11 (37.9) |
| 1 | 16 (55.2) |
| 2 | 2 (6.9) |
| Hypercreatinemia |  |
| 0 | 28 (96.6) |
| 1 | 0 (0) |
| 2 | 1 (3.4) |
| Hyperbilirubinemia |  |
| 0 | 25 (86.2) |
| 1 | 4 (13.8) |
| Insomnia |  |
| 0 | 28 (96.6) |
| 1 | 1 (3.4) |
| PT prolonged |  |
| 0 | 27 (93.1) |
| 1 | 2 (6.9) |
| Pain |  |
| 0 | 18 (62.1) |
| 1 | 10 (34.5) |
| 2 | 1 (3.4) |
| Vomiting |  |
| 0 | 17 (58.6) |
| 1 | 9 (31.0) |
| 2 | 3 (10.3) |
| Fever |  |
| 0 | 27 (93.1) |
| 1 | 1 (3.4) |
| 2 | 1 (3.4) |
| Hypertension |  |
| 0 | 27 (93.1) |
| 1 | 2 (6.8) |
| Constipation |  |
| 0 | 28 (96.6) |
| 1 | 1 (3.4) |
